# Sun Exposure Prevalence and Associated Skin Health Habits: Results from the Austrian Population-Based UVSkinRisk Survey

**DOI:** 10.3390/ijerph13010141

**Published:** 2016-01-19

**Authors:** Daniela Haluza, Stana Simic, Hanns Moshammer

**Affiliations:** 1 Institute of Environmental Health, Center for Public Health, Medical University of Vienna, Kinderspitalgasse 15, Vienna A-1090, Austria; hanns.moshammer@meduniwien.ac.at; 2 Institute of Meteorology, University of Natural Resources and Life Sciences, Vienna, Peter-Jordan-Straße 82, Vienna A-1190, Austria; stana.simic@boku.ac.at

**Keywords:** public health, preventive medicine, sunlight exposure, photo-protective behavior

## Abstract

Recreational sun exposure accounts for a large number of acute and chronic dermatological diseases, including skin cancer. This study aimed at estimating the one-year prevalence of sun exposure and skin health-associated knowledge and attitudes among Austrian citizens. The population-based UVSkinRisk survey investigated a representative sample of Austrian adults using a structured questionnaire. In total, 1500 study subjects (median age 33.0 years, 50.5% females) participated in this questionnaire survey. Among study participants, prevalence of sun exposure was 47%, with slightly higher rates in males (48%) compared to females (46%). Younger age, lower professional category, darker skin type, motives to tan, sunbed use, sunburn, and outdoor sport activity increased the odds for prevalent sun exposure. This is the first population-based study evaluating the prevailing sun exposure and recreational habits influencing skin health among Austrian citizens. Despite public media campaigns educating on the harmful effects of sunlight exposure, we found a high prevalence of self-reported sunlight exposure. The results suggest that multifaceted socio-cultural characteristics stimulate recreational sun exposure and tanning habits. Communicating individualized Public (Skin) Health messages might be the key to prevent photo-induced skin health hazards in light-skinned populations. The practical and theoretical implications of these findings are discussed.

## 1. Introduction

Incidence rates of melanoma and nonmelanoma skin cancer are high and still increasing in fair-skinned populations throughout the world [1]. Skin malignancies comprise the most common cancers and hence, their treatment and surveillance require huge healthcare expenses [2]. Relative to population size, annual direct health system costs for skin cancer are highest for Australia, New Zealand, Sweden, and Denmark [3]. Skin cancers are mainly caused by recreational intermittent ultraviolet (UV) light exposure such as outdoor activities [1]. Thus, disease prevention is feasible simply by sun avoidance or appropriate photo-protection [4]. In view of the current demographic changes and rising skin cancer incidence rates, health economic analyses of skin health promotion campaigns imply cost effectiveness, if not cost saving [5].

In spite of ongoing efforts to educate on respective acute and chronic health hazards, public concerns [6,7] regarding potential risks of artificial and natural UV radiation (UVR) exposure are still undervalued [7,8]. Therefore, it is critical to understand how target group-specific health beliefs and behavior correlate with socio-demographic characteristics. Research that fosters the understanding of common sun safe practices provides the scientific evidence to identify relevant targets for more effective “safe fun in the sun” strategies.

Recently, we introduced the umbrella term Public (Skin) Health for Public Health endeavors addressing lifestyle habits affecting skin health [9,10,11,12,13,14]. Based on the definition of the broader concept of Public Health, Public (Skin) Health refers to public and private measures to prevent skin disease, promote skin health, and monitor populations at risk in order to identify local and national health priorities. The associated research activities should detect risk groups and facilitate provision of evidence-based educative information and formulation of public policies. As Public (Skin) Health strives at encouraging skin health practices, the outcome of skin health promotion among the population is primarily reflected in lower incidence rates of photo-induced skin manifestations [15].

National differences regarding melanoma prognosis across the European continent suggest the need for studying country-specific skin health habits [1]. A considerable amount of studies have investigated these aspects in other European countries, e.g., Sweden [16] and France [17,18]. Yet, little is known on potential lifestyle-associated explanations for rising melanoma incidence and mortality rates in Austria [19]. Thus, we conducted the population-based UVSkinRisk survey to investigate the prevalence of sun exposure of Austrian citizens and factors associated with sun exposure and photo-protective behaviors [9,10,14,19]. The practical and theoretical implications of these findings for future Public (Skin) Health campaigns and doctor-patient counseling are discussed.

## 2. Methods

### 2.1. Study Population and Data Collection

The current study evaluated cross-sectional data of 1500 Austrian citizens collected by the population-based UVSkinRisk survey [9,10,14,19]. The German-speaking Austrian adult population comprised the target population of this study. Thus, the quota method ensured that the gender-balanced study sample selected by stratified random sampling represented the Austrian population in terms of age, population of federal states, and place of residence. The study design was approved by the local ethical committee of the Medical University of Vienna and conducted according to the guidelines laid down in the Declaration of Helsinki. Interviewers trained in appropriate ethics procedures obtained verbal consent from all participants prior to the telephone interviews. For data collection, we used a pre-coded, standardized study questionnaire. The surveys took approximately 10 (range 8 to 12) min to complete. Computer assisted interviewing and study questionnaire design avoided item non-response and missing data.

### 2.2. Study Measures

Each study subject provided baseline data on age, gender, place of residence, city size, highest attained education, occupational and personal situation, smoking status, sport activity, skin type as well as personal and family history of skin cancer. We collected information on current individual smoking habits as well as regular outdoor sport activity by the dichotomous questions (no/yes) “Do you smoke or have you ever smoked cigarettes?” and “Do you usually do outdoor sports?”, respectively. Further, participants classified their skin type ranging from fair (I) to dark (VI) skin pigmentation according to the Fitzpatrick Scale [8]. We assessed personal and family skin cancer history by “Have you ever been diagnosed with skin cancer?” and “Have any of your close family members ever been diagnosed with skin cancer?” respectively [20].

According to the Nomenclature of Territorial Units for Statistics (NUTS), we divided the geographic regions of Austria into the three parts East (Burgenland, Lower Austria, and Vienna), South (Carinthia and Styria), and West (Upper Austria, Salzburg, Tyrol, and Vorarlberg) [21]. We itemized current type of occupation in the socio-professional categories (SPC) SPC+ (white-collar, freelance work) and SPC− (manual work, domestic service) as well as retired/unemployed [18]. We classified variables into standard categories for education (primary, secondary, tertiary), smoking status (non-smoking, smoking), personal as well as family skin cancer history (yes, no).

We further collected self-reported information on connectedness to nature, sunburn, sun exposure and protection habits, skin health knowledge, and motives to tan. The single item operationalizing connectedness to nature used the eleven-step rating question “How would you rate your connectedness to nature?” ranging from none (=0) to very high (=10) [9]. The question “In the past year, did you receive a sunburn?” measured occurrence of sunburn in the past year (no/yes). We assessed sunbed use (no/yes) by the question “In general, do you use sunbeds?”. The question “In the past year, how many days did you sunbathe outdoors?” assessed sunbathing frequency as a measure for sun exposure during leisure time and vacation. For analytical reasons, we dichotomized sun exposure (no/yes) by assigning participants indicating 0–5 days of sun exposure to the “no sun exposure” group and those with >5 days of sun exposure to the “sun exposure” group, respectively. The question “In the past year, how long did you sunbath outdoors on a sunny day?” measured duration of sun exposure ranging from <30 min to >3 h. The question “In the past year, how long did you sunbathe outdoors during midday hours?” asked for sun exposure around noon spanning from <30 min to >4 h.

A test of seven true-false questions measured participants’ skin health knowledge related to avoidance of sun exposure during midday hours, skin cancer risk of sunbed use/sun exposure, photo-aging risk of sun exposure, and sunscreen use on pre-tanned skin/to protect photo-aging/for safe sunbathing. Summed amount of correct responses built the covariate knowledge score with higher scores indicating better knowledge. Participants provided information on frequency of using sun-protective measures (basically: “For sun protection I use sunscreen (min. SPF 15)/reapply sunscreen during the day/reapply sunscreen after swimming/avoid midday sun/seek shade/wear a hat/wear protective garments/wear sunglasses.”) by a five-point Likert scale scoring from always (=1) to never (=5). In addition, respondents rated their degree of agreement with motives to tan (basically: “A tanned skin is desirable because it enhances sex appeal/enhances attractiveness/enhances self-confidence/enhances fitness/enhances body shape/reduces paleness/reduces acne/reduces stretch marks.”) using a five-point Likert scale ranging from strongly agree (=1) to strongly disagree (=5).

Corresponding to median of respective item responses, these factors were dichotomized (low/high) to generate subgroups (connectedness to nature: low: 0–7, high: 8–10; none = 0, very high = 10; knowledge score: low: 0–4, high: 4–7; weak knowledge = 0, full knowledge = 7; motives to tan: low: 0–4, high: 4–5; fully agree = 1, fully disagree = 5; and sun protection: low: 0–3, high: 3–5; very frequently = 1, never = 5). In order to generate the covariate sun protection and motives to tan, we summed the set of scores and calculated the mean of these items. The scales motives to tan and sun protection showed an acceptable internal consistency (Cronbach’s alpha = 0.64 and 0.73, respectively), as suggested by the literature, e.g., [22,23].

### 2.3. Statistical Analysis

Data were expressed as proportions, means, and standard deviation (SD) values and continuous variables were dichotomized where appropriate. We defined specifications of motives to tan and sun protection (more/less) according to median splitting (low/high). We used the bootstrapping method based on 1000 bootstrap samples to obtain 95% confidence intervals (95% CI) as estimates for one-year prevalence of sun exposure.

We performed binary logistic regression analysis using backward stepwise elimination to predict sun exposure. To control for confounding, we calculated both crude (simple regression model) and adjusted (multiple regression model) odds ratios (OR) and 95% CI. We presented results of the adjusted model and only considered factors with a statistically significant difference in distribution as potential predictors [24]. The regression model included the variables education (primary *vs.* higher), age (younger *vs.* older age), socio-professional category (SPC+ *vs.* SPC− and unemployed/retired), personal status (single *vs.* other domestic situations), sunbed use, sunburn and sport activity (all yes *vs.* no), motives to tan (more *vs.* less), and skin type (fair *vs.* darker skin types). For all statistical analyses, we used SPSS Version 21.0 (SPSS Inc., Chicago, IL, USA) and set the two-sided level of significance at *p* = 0.05.

## 3. Results

In total, 1500 study subjects (50.5% females) participated in this population-based questionnaire survey. Median age of participants was 33.0 years (range: 25.0–44.0 years), median connectedness to nature 8.0 (6.0–9.0), median motives to tan 3.0 (2.4–3.5), median sun protection 2.6 (1.9–3.3), and median skin health knowledge 4.0 (4.0–5.0), respectively, also see [9]. Table 1 shows the gender-specific prevalence regarding sun exposure frequency and duration in general and during noon. Whereas frequency of sun exposure did not differ statistically significantly between genders, male participants reported longer duration in general and during noon (both *p* < 0.050).

**Table 1:** Sun exposure prevalence (frequency, duration, and during midday hours), stratified by gender.

Factor	Total		Females		Males		*P* (chi^2^)
	N	%	N	%	N	%	
Total	1500	100	758	100	742	100	
**Frequency of sun exposure ^a^**							
Never	506	33.7	277	36.5	229	30.9	
1–5 days	287	19.1	130	17.2	157	21.2	
6–15 days	302	20.1	154	20.3	148	19.9	
16–30 days	254	16.9	126	16.6	128	17.3	
>30 days	151	10.1	71	9.4	80	10.8	0.108
**Duration of sun exposure ^b^**							
Never	506	33.7	277	36.5	229	30.9	
<30 min	204	13.6	109	14.4	95	12.8	
30–60 min	227	15.1	112	14.8	115	15.5	
1–3 h	337	22.5	165	21.8	172	23.2	
30–60 min	226	15.1	95	12.5	131	17.7	0.024 *
**Sun exposure during midday hours ^c^**							
Never	506	33.7	277	36.5	229	30.9	
<30 min	161	10.7	90	11.9	71	9.6	
30–60 min	295	19.7	140	18.5	155	20.9	
1–2 h	280	18.7	140	18.5	140	18.9	
2–4 h	160	10.7	70	9.2	90	12.1	
>4 h	98	6.5	41	5.4	57	7.7	0.029 *

Notes: * *p* < 0.05 for gender differences (chi^2^); **^a^** In the past year, how many days did you sunbathe outdoors? **^b^** In the past year, how long did you sunbathe outdoors on a sunny day? **^c^** In the past year, how long did you sunbathe outdoors during midday hours?

Table 2 shows basic characteristics of the study population stratified by dichotomized one-year sun exposure prevalence (no/yes): 0–5 days per year (no sun exposure) and >5 days per year (sun exposure). In our sample, overall one-year prevalence of sun exposure (dependent variable) was 47.1% (95% CI 44.6–49.7, *n* = 707), with slightly higher rates in males (48.0%, 95% CI 44.5–51.3, *n* = 356) compared to females (46.3%, 95% CI 42.9–49.9, *n* = 351). Regarding socio-demographic characteristics, sun exposure prevalence statistically significantly decreased with age, was highest in younger ages (18–49 years: 60.3%–51.2%) and when living with a child (51.7%–50.3%) or being divorced, widowed *etc.* (58%), all *p* < 0.001, and increased with education level (50.1% among higher educated study participants, *p* = 0.039).

Accordingly, Table 3 presents distributions of personal recreational skin risk behavior and factors stratified by sun exposure prevalence (no/yes). Prevalences differed statistically significantly between participants who reported sun exposure and those who did not for skin types IV–VI (56.7%), sunbed use (65.7%), occurrence of sunburn (60.3%), sport activity (53.1%), more motives to tan (66.7%), all *p* < 0.001, as well as lack of family skin cancer history (48.0%, *p* = 0.033).

To further scrutinize the aforementioned findings, we performed a binary linear regression analysis including variables that statistically significant differed between participants with and without sun exposure shown in Table 2 and Table 3 (Table 4). Nagelkerke’s R^2^ suggested that the model explained roughly 19% of the variance in the outcome and that the model was a good fit to the actual data (*p* > 0.05), as did the Hosmer-Lemeshow goodness-of-fit test (chi^2^ = 7.590, *p* = 0.474).

**Table 2:** Socio-demographic characteristics of the study population, stratified by sun exposure prevalence (no/yes).

Factor	N	No Sun Exposure ^†^		Sun Exposure ^†^		*P* (chi^2^)
		N	%	N	%	
**Gender**						
Female	758	407	53.7	351	46.3	
Male	742	386	52.0	356	48.0	0.517
**Age (years)**						
18–29	305	121	39.7	184	60.3	
30–39	278	120	43.2	158	56.8	
40–49	340	166	48.8	174	51.2	
50–59	260	161	61.9	99	38.1	
60–74	317	225	71.0	92	29.0	0.001 **
**Geographic regions of Austria**						
East	644	352	54.7	292	45.3	
South	320	176	55.0	144	45.0	
West	536	265	49.4	271	50.6	0.139
**Place of residence**						
Rural < 2000 inhabitants	301	157	52.2	144	47.8	
Rural < 5000 inhabitants	345	192	55.7	153	44.3	
City < 50,000 inhabitants	374	194	51.9	180	48.1	
City > 50,000 inhabitants	480	250	52.1	230	47.9	0.705
**Education level**						
Primary	357	209	58.5	148	41.5	
Secondary	706	366	51.8	340	48.2	
Tertiary	437	218	49.9	219	50.1	0.039 *
**Socio-professional category**						
SPC+	572	280	49.0	292	51.0	
SPC−	475	240	50.5	235	49.5	
Retired/unemployed	453	273	60.3	180	39.7	0.001 **
**Personal status**						
Single	247	148	59.9	99	40.1	
Partner	371	224	60.4	147	39.6	
Single with child	58	28	48.3	30	51.7	
Partner with child	622	309	49.7	313	50.3	
Other (divorced, widowed *etc.*)	202	84	41.6	118	58.4	0.001 **

Notes: * *p* < 0.05; ** *p* < 0.001; ^†^ One-year sun exposure prevalence: 0–5 days per year (no sun exposure) and >5 days per year (sun exposure).

The variables age, socio-professional category, skin type, sunbed use, sunburn occurrence, regular sport activity, and motives to tan predicted prevalent sun exposure. The socio-economic features age (decreasing with age by trend, OR= 2.0–3.7, 95% CI 1.2–6.2) and SPC (SPC− and retired/unemployed compared to SPC+, OR = 1.8, 95% CI 1.2–2.8) predicted sun exposure. Also, skin type (increasing with pigmentation, OR = 2.2–3.6, 95% CI 1.2–6.6), motives to tan (OR = 2.4, 95% CI 1.9–3.0) as well as the recreational habits sunbed use (OR = 1.6, 95% CI 1.1–2.3) and outdoor sport activity (OR = 1.6, 95% CI 1.3–2.1) as well as sunburn occurrence (OR = 1.7, 95% CI 1.3–2.2) increased the odds for prevalent sun exposure. Noteworthy, education level, family status and family skin cancer did not predict sun exposure.

**Table 3:** Skin health characteristics of the study population, stratified by sun exposure prevalence (no/yes).

Factor	N	No Sun Exposure ^†^		Sun Exposure ^†^		*P* (chi^2^)
		N	%	N	%	
**Smoking**						
Yes	659	357	54.2	302	45.8	
No	841	436	51.8	405	48.2	0.370
**Skin type**						
I	79	59	74.7	20	25.3	
II	441	239	54.2	202	45.8	
III	657	355	54.0	302	46.0	
IV–VI	323	140	43.3	183	56.7	0.001 **
**Skin cancer history**						
**Personal**						
Yes	230	115	50.0	115	50.0	
No	1270	678	53.4	592	46.6	0.344
**Family**						
Yes	147	90	61.2	57	38.8	
No	1353	703	52.0	650	48.0	0.033 *
**Sunbed use**						
Yes	134	46	34.3	88	65.7	
No	1366	747	54.7	619	45.3	0.001 **
**Sunburn**						
Yes	461	183	39.7	278	60.3	
No	1039	610	58.7	429	41.3	0.001 **
**Sport activity**						
Yes	942	442	46.9	500	53.1	
No	558	351	62.9	207	37.1	0.001 **
**Connectedness to nature ^a^**						
Low	475	242	50.9	233	49.1	
High	1025	551	53.8	474	46.2	0.311
**Knowledge ^b^**						
Low	319	160	50.2	159	49.8	
High	1181	633	53.6	548	46.4	0.275
**Motives to tan ^c^**						
Low	732	350	47.8	488	66.7	
High	768	443	57.7	219	28.5	0.001 **
**Sun protection ^d^**						
Low	722	380	52.6	342	47.4	
High	778	413	53.1	365	46.9	0.861

Notes: * *p* < 0.05; ** *p* < 0.001; ^†^ One-year sun exposure prevalence: 0–5 days per year (no sun exposure) and >5 days per year (sun exposure); **^a^** Connectedness to nature: low: 0–7, high: 8–10; none = 0, very high = 10; **^b^** Knowledge score: low: 0–4, high: 4–7; weak knowledge = 0, full knowledge = 7; **^c^** Motives to tan: low: 0–4, high: 4–5; fully agree = 1, fully disagree = 5; **^d^** Sun protection: low: 0–3, high: 3–5; very frequently = 1, never = 5).

**Table 4:** Results of the linear regression analysis on sun exposure prevalence.

Factor	OR	95% CI	*p*-Value
**Education**			
Primary	1	-	0.610
Secondary	1.137	0.850–1.521	0.387
Tertiary	1.190	0.824–1.72	0.354
**Age**			
18–29	1	-	0.001 **
30–39	3.570	2.108–6.046	0.001 **
40–49	3.630	2.143–6.149	0.001 **
50–59	2.944	1.773–4.887	0.001 **
60–74	1.949	1.213–3.131	0.006 *
**Socio-professional category**			
SPC+	1	-	0.020 *
SPC−	1.120	0.820–1.530	0.475
Retired/unemployed	1.829	1.194–2.8	0.005 *
**Personal status**			
Single	1	-	0.503
Partner	1.136	0.709–1.821	0.595
Single with child	1.119	0.715–1.751	0.624
Partner with child	1.336	0.673–2.651	0.408
Other	1.397	0.892–2.187	0.144
**Sunbed use (yes)**	1.590	1.066–2.371	0.023 *
**Sunburn (yes)**	1.698	1.318–2.187	0.001 **
**Motives to tan (more)**	2.380	1.904–2.975	0.001 **
**Skin type**			
I	1	-	0.001 **
II	2.207	1.243–3.921	0.007 *
III	2.416	1.373–4.254	0.002 *
IV–VI	3.664	2.028–6.62	0.000 **
**Family skin cancer (yes)**	1.168	0.858–1.591	0.324
**Sport activity (yes)**	1.638	1.297–2.069	0.001 **

Notes: * *p* < 0.05; ** *p* < 0.001.

## 4. Discussion

The present cross-sectional study analyzed data collected by the UVSkinRisk survey [9,10,14]. This survey aimed at providing so far lacking empirical data on lifestyle-related sun exposure habits among a sample representing the Austrian socio-demographic population in terms of age and place of residence. Overall one-year sun exposure prevalence of 47% was quite high, with slightly higher rates in males (48%) compared to females (46%). Duquia *et al.* found lower sun exposure prevalences among Brazilian men and women when studying sun exposure during midday hours at the beach (males: 33%, females: 26%) and at work (40% and 11%), respectively [25]. In a French study, 78% of study subjects exposed themselves to the sun, with as many as 38% of them did not use appropriate sun protection measures [17]. In the current study, frequency of sun exposure did not differ statistically significantly between genders, whereas male participants reported longer duration in general and during noon (both *p* < 0.050).

In our adjusted regression model, younger age, lower socio-professional category, darker skin type, sunbed use, occurrence of sunburn, more motives to tan, and regular sport activity predicted prevalent sun exposure. The variables education level, family status as well as family skin cancer did not predict sun exposure. In the aforementioned Brazilian study sample, sun exposure was not associated with self-reported skin color, but with family income and achieved schooling [25]. However, gender, age, educational level and skin type were the most influencing factors affecting sun exposure and sun protection habits among Swedish study participants [26].

High UVR exposure prevalences reported worldwide associated with a vast variety of socio-cultural characteristics that stimulate recreational sun exposure and tanning habits suggest that customized Public (Skin) Health messages might be the key to prevent photo-induced skin health hazards. Our study results on quite high sun exposure prevalence suggest that sun avoidance seems no option for many individuals in Austria. This might arise from the notion that a tanned skin is still perceived as attractive and UVR exposure stimulates mood and wellbeing, but also as awareness of an immediate health risk is missing comparable to cigarette smoking [6]. Given the high healthcare costs associated with treatment of photo-induced skin damage, evaluating country-specific recreational habits contribute to understand how to motivate and empower individuals towards sustainable skin health promotion [2].

Appearance reasons to tan (e.g., enhancement of physical attractiveness) and not to tan (long-term: skin aging, short-term: sunburn) as well as socio-cultural influences by media, friends, and family were identified as main motives to tan [27]. Cafri *et al.* suggested using psychosocial health behavior models to predict sunbathing intentions mediated by the relationship between appearance attitudes and tanning behaviors and the perceived skin health risk [28]. Skin health campaigns that deliberately address these social and psychological barriers associated with non-uptake of sun protection might be more effective in behavior change [6]. This would comply with our findings that skin health knowledge per se is not changing photo-protection behavior, as also shown by other authors [29].

Conventional skin health campaigns usually appeal to a limited part of the general public and often fail to encourage a healthy lifestyle among risk groups [6]. Targeted awareness campaigns could be effective in promoting reasonable sun exposure habits, e.g., among younger individuals. We assume that this age group is at risk for unprotected sun exposure due to being more prone to mass media content as well as peer pressure influencing their motives to tan and recreational sun exposure habits [30,31]. By presenting conflicting and biased health messages, the mass media seems not to be an appropriate means for promoting sun protection strategies [11,32,33]. In nowadays information societies, Internet-based tools for communication and health promotion offer innovative melanoma prevention strategies [34,35,36]. Online, interactive, educational programs could potentially enhance public participation in skin health promotion, especially meeting the younger population on their level.

In our study, occurrence of sunburn as an important short-term marker for excessive sun exposure predicted sun exposure. Likewise, risk of melanoma is increasing with the number of sunburns during one’s lifespan, not just in childhood [37]. Davis *et al.* also found that summer sunburn was the norm among U.S. youths [33]. Accordingly, the majority of the U.S. population engages in numerous skin cancer risk behaviors such as infrequent sunscreen use, especially among younger, male, and less-educated participants [38]. Heckman *et al.* reported on the importance of psychosocial correlates of sunburn including fair skin, higher perceived susceptibility to skin cancer, and also greater perceived benefits of tanning among female undergraduate university students in the U.S. [39]. From a Public (Skin) Health perspective, sustained reduction of blistering sunburns during all life periods could be a measure of the effectiveness of skin cancer prevention efforts. Given the latency period for skin cancer induction by UVR exposure, these aspects might be especially relevant in view of aging societies.

## 5. Strengths and Limitations

The present study is the first empirical approach that collected data from a large, community-based sample with the study participants’ age and gender distributions representing the Austrian census data [10]. Amount of participants (*n* = 1500) was comparable to similar European studies such as the French EDIFICE Melanoma survey (*n* = 1502) [17,18]. Other respective skin health studies such as Falk *et al.* included even fewer participants (*n* = 415) [26]. We believe that Public (Skin) Health research has wide implications for clinical practice and community skin health promotion. Closing the knowledge gap regarding recreational skin health habits in Austria, the current study theoretically advances the understanding of prevailing sun exposure habits in the general Austrian population. Thus, the herein presented data may serve as a valuable baseline for tracking progress achieved by future Public (Skin) Health campaigns [33].

Questionnaire-based surveys assessing self-reported data are commonly used studying both past and current sun exposure and protection habits at population level. However, some limitations of this research approach should be acknowledged. Self-reported data that rely on retrospective information introduce unavoidable potential recall bias. In addition, social, cultural or educational factors can influence individual responses. This study design’s advantages comprise the ability to collect data on non-observable factors influencing behaviors such as skin health knowledge and attitudes [15].

Individual sun exposure is challenging to measure, particularly for diseases with substantial latency periods between first exposure and diagnosis of dermatological malignancies. Thus, collecting environmental information such as latitude and weather data as surrogates of personal UVR related to location of residence could serve as surrogate for personal long-term sun exposure [19,40]. In line with other authors, we divided the study population in two groups of “sun exposure” and “no sun exposure” to distinguish between people receiving a considerable amount of UVR or not by using a dichotomized variable [17,18]. As this study focused on recreational sun exposure and associated sun-seeking behavior, this analysis did not consider other factors that potentially influence the actual amount of sun exposure including occupational sun (over)exposure.

Self-reported and observed data on sun protection compliance have been shown to be concordant even over a longer time period [15]. Also, Parr *et al.* showed a small recall bias for sun exposure [41]. Thus, we consider that self-reported information is suitable to assess a population’s recreational skin health habits. De Waal and co-workers found evidence for reproducibility of self-reported phenotypic characteristics, sunburn history, sun exposure and photo-protection [42]. As these authors suggest the usefulness of questionnaire data for these research approaches, we assume that our data represent a reliable picture of the actual skin health habits displayed by the Austrian population.

## 6. Conclusions

The present study evaluates recreational skin health habits among the Austrian population to identify strategies how to motivate and empower individuals towards sustainable skin health promotion. Despite public media campaigns educating on the harmful effects of sunlight exposure, we found a high prevalence of self-reported sunlight exposure. The findings of this study further suggest that multifaceted socio-cultural characteristics including age and occupation stimulate leisure time sun exposure and intentional tanning. Communicating target-group sensitive Public (Skin) Health messages might be the key to prevent photo-induced skin diseases in light-skinned populations.
